# Back into the wild—Apply untapped genetic diversity of wild relatives for crop improvement

**DOI:** 10.1111/eva.12434

**Published:** 2016-12-10

**Authors:** Hengyou Zhang, Neha Mittal, Larry J. Leamy, Oz Barazani, Bao‐Hua Song

**Affiliations:** ^1^ Department of Biological SciencesUniversity of North Carolina at CharlotteCharlotteNCUSA; ^2^ The Institute for Plant SciencesIsrael Plant Gene BankAgricultural Research OrganizationBet DaganIsrael

**Keywords:** advanced biotechnology, climate change, conservation, crop wild relatives, environmental stresses, food security

## Abstract

Deleterious effects of climate change and human activities, as well as diverse environmental stresses, present critical challenges to food production and the maintenance of natural diversity. These challenges may be met by the development of novel crop varieties with increased biotic or abiotic resistance that enables them to thrive in marginal lands. However, considering the diverse interactions between crops and environmental factors, it is surprising that evolutionary principles have been underexploited in addressing these food and environmental challenges. Compared with domesticated cultivars, crop wild relatives (CWRs) have been challenged in natural environments for thousands of years and maintain a much higher level of genetic diversity. In this review, we highlight the significance of CWRs for crop improvement by providing examples of CWRs that have been used to increase biotic and abiotic stress resistance/tolerance and overall yield in various crop species. We also discuss the surge of advanced biotechnologies, such as next‐generation sequencing technologies and omics, with particular emphasis on how they have facilitated gene discovery in CWRs. We end the review by discussing the available resources and conservation of CWRs, including the urgent need for CWR prioritization and collection to ensure continuous crop improvement for food sustainability.

## 1 Introduction

Global climate change, human activities, population expansion, plant colonization, and increasing competition for land, water, and energy are the key challenges confronting food production in the 21st century (Godfray et al., 2010). Rising global temperatures are expected to have broad environmental effects such as altered patterns of drought and salinity and the emergence of new pests and diseases that will adversely impact plant growth and yield (Tester & Langridge, 2010). It also has been suggested that changes in atmospheric CO_2_ may negatively affect biodiversity and endanger crop productivity by stimulating the growth of invasive weeds (Raizada, Singh, & Raghubanshi, 2009). Moreover, the world population is predicted to reach over 9 billion by the year 2050 (U.S. Bureau of the Census 2016), and feeding this fast‐growing population is generating increased pressure on agricultural crop production (Dempewolf et al., 2014; Kastner, Rivas, Koch, & Nonhebel, 2012; Khoury et al., 2014). To maintain or increase food supply to meet present and future challenges, it is essential that we develop new crop varieties with increased tolerance/resistance to environmental stresses.

**Figure 1:**
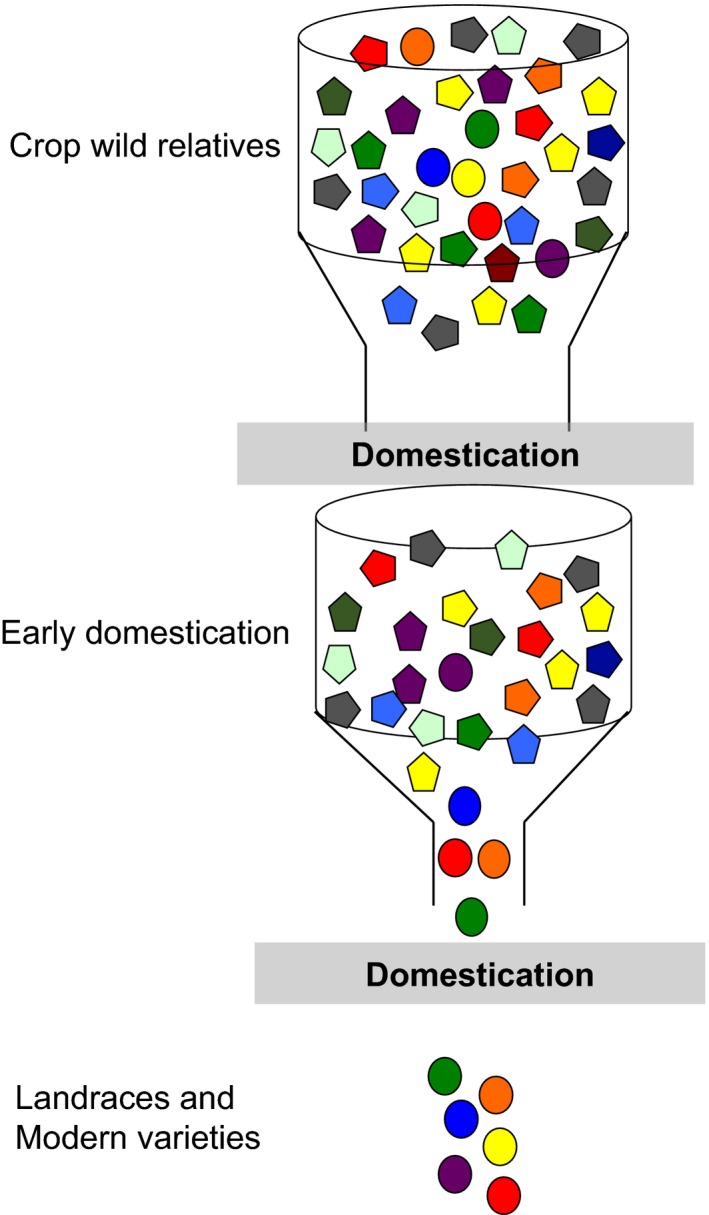
The decrease in genetic diversity in modern crops during domestication due to bottleneck events

Plant domestication is an evolutionary process in which humans have used wild species to develop new and altered forms of plants with morphological or physiological traits that meet human needs. Typically, limited numbers of individuals of progenitor species were used by early farmers and the traits selected usually were related to overall yield, harvesting, and edibility (Hua et al., 2015; Konishi et al., 2006). As a consequence, this strong selection process produced genetic bottlenecks of varying degrees that have resulted in a heterogeneous reduction in the level of genetic variation among annual herbaceous crops (Figure 1) (Buckler, Thornsberry, & Kresovich, 2001; Meyer, DuVal, & Jensen, 2012; Miller & Gross, 2011).

The domestication process has resulted in reduced diversity at both the genome and local levels. For example, more than half of the genetic variation has been lost in cultivated soybean (Hyten et al., 2006; Zhou et al., 2015), 2–4% of maize genes experienced artificial selection (Wright et al., 2005), and genetic diversity has been significantly reduced in cultivated rice (Xu et al., 2012), as compared with its wild counterpart (Figure S1). In addition, positive selection on a target locus controlling a domestication trait of interest can also result in a reduction in the diversity of closely linked loci (selective sweep). In fact, many of these selective sweeps have been found at previously reported quantitative trait loci (QTL) associated with domesticated traits. Examples include a approximately 90‐kb selective sweep at the promoter region of axillary‐branch formation‐related gene *tb1* in maize (Clark, Linton, Messing, & Doebley, 2004), a 600‐kb sweep at kernel‐related gene *Y1* in maize (Palaisa, Morgante, Tingey, & Rafalski, 2004), and a 260‐kb sweep at amylose‐related gene *waxy* in rice (Sweeney & Mccouch, 2007). These results suggest that domestication has reduced or eliminated genetic diversity at certain loci in modern crops, thus limiting to some extent their potential for developing novel varieties with improved traits. In contrast, crop wild relatives (CWRs) retain high levels of genetic diversity compared to their domesticated descendants (Figure 1). In most cases, CWRs are more diverse at both the population level and the individual level (e.g. heterozygosity) except for clonally propagated perennial crops that retain high levels of heterozygosity in domesticated lineages (McKey, Elias, Pujol, & Duputie, 2010; Zohary & Spiegelroy, 1975). Here, we focus on population‐level diversity.

There are two ways in which CWRs have been defined. One is the gene pool (GP) concept proposed by Harlan and de Wet (1971), where CWRs were classified into groups (GP‐1–GP‐3) based on the relative ease of gene exchange with cultivated crops. Gene exchange occurs relatively easily between primary (GP‐1) and secondary GPs (GP‐2) by crossing (and fertile hybrids can be produced), whereas gene transfer between primary and tertiary (GP‐3) groups is usually difficult. Even though the CWRs that have been used in crop improvement mostly belong to GP‐1 (Munns et al., 2012) or GP‐2 categories (Fetch, Johnston, & Pickering, 2009; Saintenac et al., 2013), there are some examples where useful alleles from distant wild relatives, such as GP‐3 plants, have been successfully transferred for crop improvement (Abedinia, Henry, Blakeney, & Lewin, 2000; Marais, Pretorius, Marais, & Wellings, 2003). The second concept for CWRs is that of the taxon group (TG), a system that is based on the ranking of the taxonomic hierarchy to crops (Maxted, Ford‐Lloyd, Jury, Kell, & Scholten, 2006). A TG may include a wide range of wild species that may be evolutionarily closely or distantly related to crop species within the same genus. With this concept, CWRs were defined in a range from TG1 (same species as the crop) to TG4 (different species within the same genus as the crop).

Crop wild relatives are widely distributed on all continents except for Antarctica, and many are located in Vavilov centers of diversity and adjacent regions (Castaneda‐Alvarez et al., 2016; Larson et al., 2014; Maxted & Kell, 2009). The global distribution of CWRs suggests that there are ample resources to be explored for use in plant breeding. In fact, among the approximately 50,000–60,000 total crop and CWR species, it has been estimated that 10,739 species (or even more) have a direct value for food security (Maxted & Kell, 2009). Although the number of publications discussing the use of CWRs in breeding has increased over the years and the use of CWR for crop improvement has been gradually recognized (Maxted & Kell, 2009), the exploration and utilization of the genetic diversity contained in wild relatives has lagged considerably. Over 70% of the total CWR species are in urgent need of collection and conservation in gene banks, and over 95% are insufficiently represented with respect to the full range of geographic and ecological variation in their native distributions (Castaneda‐Alvarez et al., 2016).

Most CWRs are currently threatened and/or are near extinction because of a variety of adverse effects caused by human population expansion and climate change (Ford‐Lloyd et al., 2011; Maxted et al., 2010). A recent study (Castaneda‐Alvarez et al., 2016) suggested that intensive and prioritized conservation actions should be undertaken in many geographic regions where the most critical collecting gaps occur. These areas include the Mediterranean and the Near East, western and southern Europe, South‐East and East Asia, and South America. Thus far, the Global Crop Diversity Trust (Crop Trust), the International Center for Tropical Agriculture (CIAT), and the Royal Botanic Gardens (Kew), in close collaboration with national and international agricultural research institutes, have initiated conservation efforts designed to fill these gaps. The storage of information for specific CWR taxa deposited in these databases, such as the taxon name, GP category, geographic distribution, breeding uses, prebreeding test results, and taxa holders, should be especially useful for breeders.

Various efforts have been made to collect a large number of wild species for plant breeding as the importance of CWRs was recognized by the Russian botanist Nikolai Vavilov in the early 20th century. Several review papers have been written from different perspectives to emphasize the importance of CWRs for crop improvement (Brozynska, Furtado, & Henry, 2015; Colmer, Flowers, & Munns, 2006; Ford‐Lloyd et al., 2011; Hajjar & Hodgkin, 2007; Maxted & Kell, 2009; Nevo & Chen, 2010; Porth et al., 2013; Redden et al., 2015; Warschefsky, Penmetsa, Cook, & von Wettberg, 2014; Zamir, 2001). Particularly because of the threats of climate change, the potential for drought and salt tolerance, increased disease and pest resistance, and enhanced yield in CWR species has been intensively explored and tested in breeding programs (Olsen & Wendel, 2013a,b). In addition, wild plant species that have never been domesticated have been used in the prebreeding and domestication process of crops such as blueberries and strawberries (Diamond, 2002). This suggests that besides CWRs, other wild plant species may become increasingly important for crop improvement.

Here, we highlight some successful examples of the use of CWRs in crop improvement, especially those from studies published after 2006. We also illustrate the usefulness of emerging advanced biotechnologies for gene discovery in wild relatives. The examples we provide are for annual, herbaceous crops only, and it should be noted that the strategies discussed might not be relevant for all crop types. Finally, we discuss the urgency and significance of CWR conservation. Our primary intent is to illustrate the potential of CWRs for crop improvement and to urge that more steps are needed for CWR conservation.

## 2 CWRs in Crop Improvement

Numerous efforts have been made to utilize the genetic diversity in CWRs to improve various crop species (Hajjar & Hodgkin, 2007; Maxted, Magos, & Kell, 2013; Nevo & Chen, 2010; Tanksley et al., 1996). These efforts have been concentrated primarily on certain crop species, including wheat, barley, rice, and tomatoes (Foolad & Panthee, 2012; Nevo & Chen, 2010; Xiao et al., 1996). Possible reasons for the greater use of CWRs in only certain crops include (i) cross‐compatibilities, (ii) the taxonomic relationship between crops and their close wild species, (iii) fertility in the F_1_ and subsequent progeny, (iv) availability or conservation of CWRs, (v) exploration and utilization of wild germplasms, and (vi) regional financial support based on local need and geographic distribution of CWRs (Zamir, 2001). Because of these and other reasons, the use of CWRs lags far behind its potential.

Despite a variety of difficulties in using CWRs that can occur especially with certain species, there are a number of examples of successful gene discovery and transfer of superior alleles from CWRs to domesticated crops. In these examples, classic genetic approaches such as map‐based cloning and backcrossing for gene introduction still play important roles in breeding regimes. Introgression lines also are increasingly used for genetic dissection of complex traits. Currently, advanced biotechnologies, such as next‐generation sequencing and high‐throughput phenotyping, are proving beneficial in accelerating gene discovery (Honsdorf, March, Berger, Tester, & Pillen, 2014; Qi et al., 2014). As a result, many promising genes or QTL associated with agriculturally important and stress‐related traits in CWRs have been identified and are being tested in breeding programs. A summary of these genes identified in CWRs of seven important crop species is given in Table 1. In the following paragraphs, we highlight some representative examples in which CWRs were used for abiotic and biotic stress resistance and yield improvement in their cultivated descendants.

**Table 1:** Examples of the application of CWRs for the improvement of biotic/abiotic stress resistance/tolerance/yields in major crops

Taxa		Traits^a^		
Crops	Wild relatives	Abiotic stress resistance	Biotic stress resistance	Agronomic traits
Rice	*Oryza minuta*;* Oryza rufipogon*;* Oryza australiensis*;* Porteresia coarctata*;* Oryza meridionalis*; O. a*ustraliensis*	Salt tolerance (Majee et al., 2004 ^G^; Sengupta & Majumder, 2010 ^R^; Rohini et al., 2014 ^O^); heat and cold tolerance (Baruah et al., 2009 ^R^; Scafaro, Haynes, & Atwell, 2010 ^R^; Baruah et al., 2011 ^R^); flooding tolerance (Niroula et al., 2012 ^G^)	Blast resistance (Liu, Lu, Zeng, & Wang, 2002 ^Q^; Huang, Hwang, Chiang, & Lin, 2008 ^G^; Yoshida & Miyashita, 2009 ^G^; Rahman, Khanam, Roh, & Koh, 2011 ^QI^; Lv et al., 2013 ^G^); planthopper resistance (Renganayaki et al., 2002 ^Q^; Jena, Jeung, Lee, Choi, & Brar, 2006 ^Q^; Du et al., 2009 ^QG^); bacterial leaf streak resistance (He et al., 2012 ^Q^); bacterial blight resistance (Huang, He, Shu, Li, & Zhang, 2001 ^Q^; Zhou et al., 2009 ^GI^; Zhou et al., 2011 ^GRI^; Hutin, Sabot, Ghesquiere, Koebnik, & Szurek, 2015 ^GR^); fungal diseases resistance (Jeon et al., 2008 ^G^; Eizenga, Agrama, Lee, & Jia, 2009 ^QR^)	Yield (Xie et al., 2008 ^Q^; Fu et al., 2010 ^Q^; Luo et al., 2011 ^Q^; Li et al., 2012 ^Q^; Zhu, Ellstrand, & Lu, 2012 ^GR^); heading date (Dai et al., 2012 ^Q^); fragrance (Prathepha, 2009 ^GR^); lipid components (Przybylski, Klensporf‐Pawlik, Anwar, & Rudzinska, 2009 ^RM^); grain quality (Han, Zhang, Qin, & Zhai, 2013 ^R^); multiple traits (McCouch et al., 2007 ^R^; Luo, Tian, Fu, Yang, & Sun, 2009 ^Q^; Ali, Sanchez, Yu, Lorieux, & Eizenga, 2010 ^QI^; Ammiraju et al., 2010 ^RO^; Das, Nayak, Patra, Ramakrishnan, & Krishnan, 2010 ^R^)
Barley	*Hordeum spontaneum*;* Hordeum* chilense	Drought tolerance (Nevo, Beiles, Gutterman, Storch, & Kaplan, 1984a, 1984b ^Q^; Diab et al., 2004 ^Q^; Suprunova et al., 2004 ^G^; Suprunova et al., 2007 ^G^; Chen et al., 2009 ^Q^; Chen et al., 2010 ^Q^; Nevo & Chen, 2010 ^QGR^; Zhao, Sun, Dai, Zhang, & Wu, 2010 ^R^; Chen et al., 2011 ^QG^; Honsdorf et al., 2014 ^Q^; Naz et al., 2014 ^QI^); salt tolerance (Nevo et al., 1984a, 1984b ^R^; Yan, Chen, Cheng, Nevo, & Gutterman, 2008 ^R^; Nevo & Chen, 2010 ^QGR^; Shavrukov et al., 2010 ^G^; Qiu et al., 2011 ^G^; Wu et al., 2011 ^GR^; Wu, Shen, et al., 2014 ^O^; Bahieldin et al., 2015 ^O^)	Fusarium crown rot resistance (Chen et al., 2013 ^Q^); scald resistance (Pickering et al., 2006 ^QG^); powdery mildew/leaf rust resistance (Schmalenbach et al., 2008 ^QI^); blotch‐related disease/powdery mildew/leaf scald resistance (Yun et al., 2005 ^Q^; Yun et al., 2006 ^Q^); late blight resistance (Danan, Veyrieras, & Lefebvre, 2011 ^Q^)	Maturity (Danan et al., 2011 ^QM^); linkage map (Rodriguez‐Suarez et al., 2012 ^Q^)
Wheat	*Triticum dicoccoides*;* Triticum aegilops*;* Triticum tauschii*;* Triticum monococcum*;* Triticum urartu*,* Agropyron elongatum*; Aegilops species; *Haynaldia villosa*;* Leymus mollis*	Drought tolerance (Nevo et al., 1984a, 1984b ^R^; Reynolds, Calderini, Condon, & Rajaram, 2001 ^QR^; Rampino, Pataleo, Gerardi, Mita, & Perrotta, 2006 ^R^; Peleg et al., 2009 ^Q^; Krugman et al., 2010 ^O^; Nevo & Chen, 2010 ^Q^; Di Bianco et al., 2011 ^GR^; Lucas, Durmaz, Akpinar, & Budak, 2011 ^G^; Placido et al., 2013 ^QI^); salt tolerance (Munns, Hare, James, & Rebetzke, 2000 ^Q^; Munns, Rebetzke, Husain, James, & Hare, 2003 ^Q^; James et al., 2006 ^I^; Mguis et al., 2008 ^R^; Munns & Tester, 2008 ^QG^; Nevo & Chen, 2010 ^QR^; Shavrukov et al., 2010 ^G^; Habora, Eltayeb, Tsujimoto, & Tanaka, 2012 ^G^; James et al., 2012 ^G^; Munns et al., 2012 ^QG^); O_3_ tolerance (Biswas et al., 2008 ^R^)	Powdery mildew fungus resistance (Blanco et al., 2008 ^Q^; Ji et al., 2008 ^Q^; Schneider, Molnar, & Molnar‐Lang, 2008 ^R^; Hua et al., 2009 ^Q^; Li et al., 2009 ^Q^; Yahiaoui, Kaur, & Keller, 2009 ^G^; Ben‐David et al., 2010 ^Q^; Cao et al., 2011 ^QG^; Xie et al., 2012 ^Q^; Xue, Ji, Wang, Zhang, & Yang, 2012 ^Q^); stem rust resistance (Leonova et al., 2007 ^Q^; Marais, McCallum, & Marais, 2008 ^Q^; Liu et al., 2011 ^QI^; Rouse & Jin, 2011 ^R^; Periyannan et al., 2013 ^QG^; Saintenac et al., 2013 ^QG^); leaf rust resistance (Marais et al., 2008 ^Q^; Murphy et al., 2009 ^Q^)	Grain quality traits (Rawat et al., 2009 ^R^; Tiwari et al., 2009 ^Q^)
Soybean	*Glycine soja*;* Glycine tomentella*	Salt tolerance (Ji et al., 2010 ^G^; Tuyen, Lal, & Xu, 2010 ^Q^; Ha et al., 2013 ^Q^; Sun et al., 2013 ^G^; Tang et al., 2013 ^G^; Qi et al., 2014 ^QGO^; Sun et al., 2014 ^G^; Wu, Zhou, et al., 2014 ^R^; Xue, Zhao, Gao, & Sun, 2014 ^R^); drought tolerance (Ji et al., 2010 ^G^; Luo et al., 2013 ^G^; Tang et al., 2013 ^G^); aluminum stress tolerance (Zeng et al., 2012 ^GO^)	Nematode resistance (Winter, Shelp, Anderson, Welacky, & Rajcan, 2007 ^Q^; Kim, Hyten, Niblack, & Diers, 2011 ^Q^); aphid resistance (Hesler, 2013 ^R^)	Multiple traits (Lam et al., 2010 ^QGO^; Wang et al., 2013 ^QI^; Li et al., 2014 ^QGO^; Singh & Nelson, 2015 ^R^; Zhou et al., 2015 ^QGO^)
Tomato	*Solanum pimpinellifolium*;* Solanum chilense*;* Solanum habrochaites*;* Solanum hirsutum*;* Solanum parviflorum*;* Solanum* lycopersicon	Antioxidant activity (Melendez‐Martinez, Fraser, & Bramley, 2010 ^M^); salt tolerance (Rao et al., 2015 ^G^); drought tolerance (Arms, Bloom, & St Clair, 2015 ^Q^)	Powdery mildew resistance (Bai et al., 2003 ^Q^); fungal pathogen resistance (Jones, Thomas, Hammondkosack, Balintkurti, & Jones, 1994 ^G^; Thomas et al., 1997 ^G^); insect pest resistance (Frelichowski & Juvik, 2001 ^M^; Mirnezhad et al., 2010 ^M^); spider mite resistance (Salinas et al., 2013 ^Q^; Antonious & Snyder, 2015 ^M^); late blight resistance (Chen et al., 2014 ^Q^; Haggard & St Clair, 2015 ^Q^); begomovirus resistance (Menda et al., 2014 ^QI^); white fly/spider mite resistance (Sallaud et al., 2009 ^M^; Bleeker et al., 2011 ^QM^; Bleeker et al., 2012 ^M^); *Tomato yellow leaf curl virus* resistance (de Castro, Blanca, Diez, & Vinals, 2007 ^M^)	Yield‐related traits (Kamenetzky et al., 2010 ^OM^; Wu et al., 2015 ^QM^); leaf traits (Muir, Pease, & Moyle, 2014 ^Q^); grain quality traits (Haque, Kjaer, Rosenqvist, & Ottosen, 2015 ^M^; Ning et al., 2015 ^M^)
Potato	*Solanum paucijugum, Solanum brevicaule*,* Solanum commersonii*;* Solanum ruiz‐ceballosii*;* Solanum* bulbocastanum; *Solanum* microdontum	Cold sweetening resistance (Hamernik, Hanneman, & Jansky, 2009 ^RI^)	Potato beetle resistance (Jansky, Simon, & Spooner, 2009 ^R^; Spooner, Jansky, & Simon, 2009 ^R^); soft rot resistance (Chung, Holmquist, Spooner, & Jansky, 2011 ^R^); potato virus resistance (Cai, Spooner, & Jansky, 2011 ^R^; Duan, Richael, & Rommens, 2012 ^G^); potato late blight resistance (Sliwka et al., 2007 ^Q^; Bhaskar et al., 2008 ^G^; Hein et al., 2009 ^Q^; Sliwka et al., 2012 ^Q^; Khiutti, Spooner, Jansky, & Halterman, 2015 ^R^; Tiwari et al., 2015 ^G^); nematode resistance (Brown, Zhang, & Mojtahedi, 2014 ^QR^); pathogen resistance (Zuluaga et al., 2015 ^O^); tuber moth resistance (Horgan, Quiring, Lagnaoui, & Pelletier, 2007 ^R^); other pests resistance (Le Roux et al., 2008 ^R^)	Multiple traits (Jansky, 2011 ^R^)
Peanut	Arachis stenosperma; Arachis duranensis: Arachis ipaënsis	Drought and fungal resistance (Guimaraes et al., 2012 ^O^)	Nematode resistance(Chu, Holbrook, Timper, & Ozias‐Akins, 2007 ^Q^; Guimarães et al., 2010 ^O^)	Multiple traits (Fonceka et al., 2012 ^QI^)

^a^Methodologies used in cited studies.

^Q^Quantitative trait loci mapping.

^G^Gene identification.

^R^Resources evaluation.

^O^Genomics/transcriptomics/proteomics.

^M^Metabolomics.

^I^Advanced backcrossing introgression lines.

### 2.1 Abiotic stress tolerance

Salinity and drought are two of the most important environmental factors limiting worldwide crop yields. The effects of both stressors have been intensely studied in primary crop species such as soybeans, tomatoes, and cereals as well as in their wild relatives (Munns et al., 2012; Placido et al., 2013; Qi et al., 2014) (Table 1). Here, we discuss several examples of the use of wild relatives for salt tolerance in wheat and soybeans, and for drought tolerance in barley.

In 2013, wheat was the third ‐most‐ produced cereal after maize and rice (“FAOStat,” retrieved January 27, 2015), and it is significantly affected by salinity (Mujeeb‐Kazi & De Leon, 2002). Intensive efforts have been made to search for salt‐tolerant genes/alleles in wheat wild relatives (Colmer et al., 2006; Nevo & Chen, 2010). Recently, Australian scientists have produced a salt‐tolerant commercial durum wheat variety by introducing an allele from its wild relative, *Triticum monococcum*, via crossbreeding. This cultivar showed 25% greater yield in high‐saline fields compared to its Tamaroi parent (Munns et al., 2012). The gene transferred was *TmHKT1;5‐A*, and has been found in a wild wheat ancestor, *Triticum monococcum* (James, Davenport, & Munns, 2006)*. TmHKT1;5‐A* reduces the Na^+^ level in plant leaves that prevents yield losses under salinity stress. This gene transfer has provided a successful example of the use of a CWR to improve crop salt tolerance. This gene could also be transferred to other wheat cultivars, such as bread wheat, to develop salt‐tolerant commercial lines. Soybean is moderately salt‐sensitive, and all developmental stages can be affected by salinity stress (Munns & Tester, 2008) that can result in a decrease in yield as high as 40% (Chang, Chen, Shao, & Wan, 1994). A salt‐tolerant gene, *GmCHX1,* was recently identified in *Glycine soja* (Sieb. & Zucc), the wild progenitor of cultivated soybean (*Glycine max* (L.) Merr.) (Qi et al., 2014). The discovery of *GmCHX1* and another salt‐tolerant gene, *GmSALT3* (Guan et al., 2014), suggests that salt‐tolerant alleles might have been lost in soybean during domestication. Both of these salt‐tolerant genes are involved in regulating ion homeostasis and offer great potential for the development of commercial soybean varieties with improved salt tolerance.

Drought is another important factor limiting crop production, especially in the context of global climate change. Although several drought‐tolerant QTL or genes have been identified in wild relatives of crops such as barley (Diab et al., 2004; Suprunova et al., 2004, 2007), wheat (Placido et al., 2013), and tomatoes (Fischer, Steige, Stephan, & Mboup, 2013), applications of these exotic genetic resources for improved drought tolerance in crops have not proven as successful as expected. This may largely be because drought tolerance is a polygenic quantitative trait presumably controlled by many QTL, each with small effects. On the other hand, the development of advanced backcross introgression libraries (ILs) provides a useful alternative method for the transfer of drought‐tolerant genes. Recently, significant progress was made on barley drought tolerance using wild barley (*Hordeum spontaneum*)‐introgressed ILs (Honsdorf et al., 2014; Naz, Arifuzzaman, Muzammil, Pillen, & Leon, 2014; Schmalenbach, Korber, & Pillen, 2008; von Korff, Wang, Leon, & Pillen, 2004). Follow‐up studies demonstrated that the ILs containing certain drought‐tolerant QTL exhibited drought tolerance in field trials (Arbelaez et al., 2015; Honsdorf et al., 2014). However, construction of ILs and exclusion of “linkage drag” genes require years of backcrossing and selection. Despite these difficulties, this approach may be our best strategy if no other effective breeding alternatives are available.

### 2.2 Biotic stress tolerance

Beyond abiotic stresses, plant yields are also reduced by attacks from various biotic stressors such as pathogens (fungi, viruses, and bacteria), nematodes, and insect pests. Even though resistant varieties have been developed, continuous use of limited numbers of resistant resources is not a long‐term strategy because pathogens and insects evolve very rapidly. To mitigate this evolutionary arms race, plant researchers and breeders have been exploiting exotic genetic resources, such as CWRs, to develop biotic stress‐resistant varieties (Hajjar & Hodgkin, 2007). These efforts include attempts to identify resistant genes in CWRs using various strategies such as metabolomics (see *Metabolomics* section) and transcriptomics. Once identified, the intent then would be to pyramid multiple exotic resistant genes into crop varieties to achieve a durable or broad‐spectrum resistance.

In maize, the corn blight of 1978 reduced the yield of corn by as much as 50% in the United States (Food and Agriculture Organization of the United Nations 2005). This was resolved by transferring blight‐resistant alleles from a wild relative of Mexican maize (*Tripsacum dactyloides* L.) into commercial corn lines (Maxted & Kell, 2009). Another devastating pest of corn in the United States is rootworm. Prischmann, Dashiell, Schneider, and Eubanks (2009) introduced genes from gama grass (*Tripsacum dactyloides* L.), a wild relative of maize that exhibits rootworm resistance, into cultivated corn. Repeated field trials showed that the descendants from this transfer appeared tolerant to rootworm damage, showing that this exotic allele was effective in combating rootworm problems.

Using wild relatives to improve biotic stress tolerance in cultivated rice has been very successful. A majority of the 22 wild rice species are being explored as alternate sources for resistance to bacterial blight, blast, brown planthopper attacks, and sheath blight (Table 1) (Jena, 2010). For example, bacterial leaf blight, caused by *Xanthomonas oryzae* pv. *oryzae* (*Xoo*), has been one of the most widely distributed and devastating rice diseases worldwide. The introduction of two resistant *Xa* genes (*Xa3* and *Xa*4) into rice cultivars has increased bacterial leaf blight resistance. However, the level of resistance in these cultivars has been decreasing as expected due to evolutionary changes in bacterial leaf blight. To address this, researchers have identified new bacterial blight‐resistant genes (*Xa21* and *Xa23*) in wild rice (Song et al., 1995; Zhou et al., 2011). These *Xa* genes have been used individually or in combination in bacterial leaf blight‐resistant rice breeding programs (Zhou et al., 2009), and this has led to significant successes in bacterial leaf blight management. Progress in combating bacterial leaf blight also has been achieved from introgression of a rice blast‐resistant gene, *Pi33*, from wild rice, *Oryza rufipogon* (Ballini et al., 2007), into the most used rice blast resistance variety (IR64).

The improvement of biotic resistant cultivated tomatoes (*Solanum lycopersicum*) also has benefitted significantly from the transfer of various traits from tomato wild relatives. These traits include resistance to bacteria, viruses, fungi, nematodes, and insect pests. The tomato wild relatives that have been used include *Solanum chilense* (Zamir et al., 1994), *Solanum habrochaites* (Prasanna, Kashyap, et al. 2015; Prasanna, Sinha, et al. 2015), *Solanum peruvianum* (Lanfermeijer, Warmink, & Hille, 2005; Seah, Yaghoobi, Rossi, Gleason, & Williamson, 2004)*, Solanum pennellii* (Parniske et al., 1999), and *Solanum pimpinellifolium* (Chunwongse, Chunwongse, Black, & Hanson, 2002). As one example, five *Ty* genes exhibiting varying degrees of resistance to *tomato yellow leaf curl virus* (TYLCY) were successfully introgressed into cultivated varieties (Ji, Scott, Hanson, Graham, & Maxwell, 2007; Menda et al., 2014). Pyramiding of these *Ty* genes from different wild tomatoes has contributed to durable and broad resistance to TYLCY (Kumar, Tiwari, Datta, & Singh, 2014; Vidavski, Czosnek, Gazit, Levy, & Lapidot, 2008).

In natural as well as in agricultural ecosystems, plants are usually simultaneously exposed to a combination of abiotic and biotic stressors. Because plant defense responses are controlled by different signaling pathways that act in an antagonistic or synergistic manner, exposure to multiple stressors may result in either a negative or a positive impact on plant performance (Atkinson & Urwin, 2012; Suzuki, Rivero, Shulaev, Blumwald, & Mittler, 2014). A comparative transcriptome analysis in *Arabidopsis* revealed gene expression responses to viral stimuli that were nullified when the plants were exposed to the viral stimulus plus any of several abiotic stresses such as drought or heat (Prasch & Sonnewald, 2013). Developing abiotic tolerant crops, either by classical breeding or by transgenic strategies, therefore may also produce plants that are immune to pathogens. On the other hand, it was recently shown that constitutive expression of the maize *ZmGF14‐6* gene in rice increased tolerance to drought but also increased susceptibility to pathogens (*Fusarium verticillioides* and *Magnaporthe oryzae*) (Campo et al., 2012). Therefore, considering that responses to single or combined abiotic stressors do not always foster enhanced biotic tolerance as well (Atkinson & Urwin, 2012; Mittler & Blumwald, 2010), integrated approaches most likely will be needed to improve plant tolerance to multiple stresses.

### 2.3 Improvement in yield and quality‐related traits

Compared with the great success of introducing biotic and abiotic stress resistance genes from CWRs for crop improvement (Colmer et al., 2006; Du et al., 2009; Munns et al., 2012), applications of CWRs for crop yield traits have been relatively less successful. There are exceptions, however, in which the magnitude of CWR contributions to yield improvement may be even greater than expected. For example, the use of a small‐fruited tomato ancestor (*S. pimpinellifolium*) (Eshed & Zamir, 1994) and a wild tomato species (*Solanum hirsutum*) led to a 20% increase in yield and soluble solids content, and an improvement in fruit color, in cultivated tomatoes (Tanksley et al., 1996). Similarly in rice, backcrossing a low‐yielding wild ancestor (*O. rufipogon*) into a Chinese hybrid strain increased its yield up to 17% (Tanksley & McCouch, 1997; Xie et al., 2008). Yield‐enhancing QTL have also been identified in wild relatives of diverse crops such as wheat, barley, soybeans, beans, and *Capsicum* (Swamy & Sarla, 2008), and it will be interesting to see how effective they might be when transferred into crops by traditional breeding or advanced biotechnological approaches.

It has been reported that the transfer of a QTL from *G. soja* into domesticated soybeans significantly increased the yield by about 190–235 kg/ha (Li, Pfeiffer, & Cornelius, 2007). Although introducing *G. soja* QTL into cultivated soybean lines of diverse parentages has often resulted in some unfavorable characteristics such as lodging susceptibility (Li et al., 2007), the improvement in yield and/or other important traits generally has made these efforts worthwhile.

The role of CWRs in the development of new varieties with improved fruit and grain quality has become increasingly important in recent years. The introduction of newly developed nutrition‐rich varieties is expected to play a role in the improvement of human health. For example, glucosinolates, a class of metabolites prevalent in crucifer species, are known to reduce the risk of various cancers (Dinkova‐Kostova & Kostov, 2012). These metabolites can easily be increased in some food plants such as broccoli where crosses between wild and cultivated broccoli produce a hybrid that contains three times more glucosinolates than conventional varieties (Sarikamis et al., 2006).

As an alternative to targeting only yield traits, researchers have incorporated abiotic and biotic stress‐tolerant traits from CWRs to achieve crop yield enhancement. Typically, crop varieties introduced with wild resistant/tolerant alleles behave similarly compared to their cultivated counterparts, but benefit when exposed to environmental stresses. For example, field trials of the wheat cultivar Tamaroi carrying the wild salt‐tolerant gene *TmHKT1;5‐A* produced yields similar to the Tamaroi parent lacking this gene when grown in fields with less saline, but produced superior yields in high‐saline fields (Munns et al., 2012). In a recent study, tomato cultivars carrying a single wild‐derived TYLCY‐resistant gene, *Ty‐2* or *Ty‐3,* or their pyramided lines, had greater yields in the presence of the pathogen TYLCY than cultivars lacking these genes (Prasanna, Kashyap, et al. 2015). The yield increases in these cases mostly are attributable to the enhanced resistance/tolerance to biotic or abiotic stresses generated by introducing the resistant/tolerant alleles from their wild relatives. Thus, the recruitment of stress resistance traits from wild relatives into modern varieties is a practical alternative for the improvement of crop yields.

## 3 Advanced Biotechnologies Accelerate the Use of Wild Relatives for Crop Improvement

The surge of diverse biotechnologies has significantly facilitated modern breeding over the past two decades (Varshney, Graner, & Sorrells, 2005). These technologies include the application of omics‐scale technologies for gene discovery, and advanced techniques to transfer genes of interest from wild plant species to cultivated crops (Table 2).

**Table 2:** Representative advanced technologies that have been used in plant breeding

Approaches	Usages	Advantages	Shortcomings	References
Genomics	Germplasm resource evaluation and identification; heterosis prediction; linkage and association mapping; marker‐assisted breeding	High‐throughput; time‐saving	Costly; bioinformatics skills required; difficulties in assembly of polyploid genomes	Brozynska et al. (2015); Langridge and Fleury (2011)
Transcriptomics and proteomics	Quantification of expression variants response to environment stress; updating genome annotation	Generating numerous candidate genes; regulatory network identification; more useful when combined with linkage analysis	Difficult to pinpoint causal genes or proteins; high cost for proteomics	Langridge and Fleury (2011); Brozynska et al. (2015)
Metabolomics	Metabolic profiling	Quantification of target or global metabolites	Costly; limited annotation data; low heritability; requiring chemical and statistical skills	Fernie and Schauer (2009)
Advanced introgression lines	Genetic mapping; introgression breeding	Traditional breeding; introducing multigenic traits	Need supports by molecular DNA markers; cross‐compatible; laborious and tedious backcrossing	Placido et al. (2013); Honsdorf et al. (2014)
Transgenesis	GM	Transfer between noncrossable species	Subject to GMO regulations; foreign genes	Schaart, van de Wiel, Lotz, and Smulders (2016)
Genome editing	GM	Precise and predefined modification	Might subject to GM regulatory regime; public acceptance	Bortesi and Fischer (2015); Schaart et al. (2016)
Cisgenesis/Intragenesis	GM	Genes from species itself or crossable species; stacking multiple genes; public acceptable; avoid linkage drag	Might require traditional breeding step	Haverkort et al. (2009); Vanblaere et al. (2011)
High‐throughput phenotyping	Phenotyping	High‐throughput; real‐time; multidimensional	High cost; mathematical and statistical skill required	Honsdorf et al. (2014); Rahaman, Chen, Gillani, Klukas, and Chen (2015)

GM, genetic modification.

*Genomic approaches* have been widely used to identify genes or genomic regions controlling complex traits. High‐throughput next‐generation sequencing technologies offer opportunities to efficiently discover SNPs associated with important traits in both diploid (Hyten et al., 2010) and polyploid plant species (Akhunov, Nicolet, & Dvorak, 2009). With recent significant cost reductions, scientists are now able to genotype thousands of individuals by genotyping‐by‐sequencing or resequencing. With the availability of increasing numbers of SNPs and phenotypic data, researchers have been able to validate and fine‐map previously identified genes and to discover novel genomic regions underlying valuable agronomic traits in CWRs by association mapping (Li et al., 2014; Qi et al., 2014; Xu et al., 2012; Zhou et al., 2015). However, the development of a high‐throughput phenotyping pipeline remains challenging, especially in the field (Kelly et al., 2016). Some of the genomic regions associated with domestication traits have enhanced our understanding of their genetic basis, and will encourage further investigation to see whether allelic variation in those regions in CWRs can additionally benefit crop improvement. Genotyping‐by‐sequencing of segregating populations (F_2_, BC2, near‐isogenic lines, and recombinant inbred lines [RILs]) allows the construction of high‐resolution linkage maps that can be used to narrow QTL regions. This strategy holds the promise of mapping QTL using fewer RILs than would be necessary with DNA markers at relatively low densities. This approach also should facilitate map‐based cloning of target genes, such as the salt‐tolerant gene *GmCHX1* unique to *G. soja* (Qi et al., 2014). It also is feasible to apply genotyping‐by‐sequencing for heterozygous plant species using case‐specific strategies (Hyma et al., 2015; Uitdewilligen et al., 2013). Thus, it is clear that the availability of this sort of genomewide data and efficient phenotyping approaches will continue to accelerate the discovery of genes controlling superior traits in CWRs.

*Other functional omics* approaches, including transcriptomics, proteomics, and metabolomics, have provided alternative opportunities for global analysis of regulatory genes, expressed proteins, or metabolite candidates underlying important traits in CWRs. These omics approaches also are particularly suitable for dissection of the variation in complex traits such as drought tolerance and pest resistance. By characterizing CWRs under diverse treatments using omics strategies, a number of stress‐resistant genes have been identified in various wild relatives of crops. For example, the dehydrin genes in both wild barley (*H. spontaneum*) and wild tomato species (*S. chilense* and *S. peruvianum*), as well as ABA/water stress/ripening‐induced (*Asr*) gene family members (*Asr2* and *Asr4*) from wild *Solanum* species, are known to be involved in drought tolerance (Fischer et al., 2013; Suprunova et al., 2004). From these and other studies with large “omics” datasets, it generally has proven difficult to pinpoint the causal genes, proteins, or metabolites underlying the traits of interest. However, this is possible when “omics” approaches are combined with other strategies such as linkage mapping (Table 1). For example, by quantifying gene expression levels within target QTL, Suprunova et al. (2007) were able to identify a novel gene (*Hsdr4*) involved in water‐stress tolerance in wild barley (*H. spontaneum*). With a similar strategy, two candidate genes (*KNAT3* and *SERK1*) conferring drought tolerance in wild wheat have also been identified (Placido et al., 2013). Further, as knowledge of transcriptome profiles under various stress conditions increases, the combination of transcriptomics, proteomics, and metabolomics with QTL analyses should prove to be a powerful tool for large‐scale study of gene function at different levels. With such an approach, however, the difference in temporal transcription and translation of genes and metabolic processes involved should be taken into account in the data interpretation (Schmollinger et al. 2014). Although the use of proteins and metabolites provides us with a deeper understanding of the mechanisms of gene function than transcriptomics alone, the high cost of metabolomics and proteomics (e.g. iTRAQ) represents a major constraint for their extensive use in plant breeding.

*Metabolomics*‐*assisted breeding* has been considered as a viable strategy for crop improvement (Fernie & Schauer, 2009). Although the high quantification cost and the relatively low levels of heritability of metabolites limit the direct application of this approach to breeding programs, several studies have revealed its potential by quantifying the variation of certain metabolites and/or metabolomics and uncovering their genetic basis with genomic approaches (Bleeker et al., 2011, 2012). Bleeker et al. (2011) showed that the application of 7‐epizingiberene, extracted from wild tomato (*S. habrochaites*) and applied on susceptible cultivated tomatoes, was effective in repelling whiteflies (Frelichowski & Juvik, 2001). The cultivated tomatoes with 7‐epizingiberene acquired from its wild relatives also showed resistance to spider mites (Bleeker et al., 2012).

Genome‐wide association studies (GWAS) of metabolomics have also become an effective way to investigate global profiles of the thousands of metabolites typically produced in plants (Luo, 2015). This approach is thought to be suitable for the exploitation of CWRs because these wild species have been subjected to long‐term evolution in their diverse natural habitats and therefore are expected to have a greater level of variation in their metabolic profiles than their cultivated descendants. CWRs do require a higher density of genomic markers for metabolomics association studies because they typically have much lower levels of linkage disequilibrium than are found in domesticated crops. Fortunately, many markers now are publically available or can be genotyped at a reduced cost. Several laboratories recently have successfully developed high‐density SNP markers for wild soybean (Song et al., 2015; Zhou et al., 2015), wild tomatoes (Aflitos et al., 2014), and wild rice (Xu et al., 2012), all of which can be or have been used in metabolomics mapping in CWRs. These and other studies (Fernie & Schauer, 2009; Schauer & Fernie, 2006) suggest that useful metabolites in CWRs can be identified and used in plant breeding. One difficulty is that most of the metabolites produced from the profiling platforms are unannotated and thus are unknown. Once identified, however, metabolite pathways can be traced by searching available annotations in a metabolomics database. It also has proven feasible to transfer an appropriate metabolite or metabolic pathway (e.g. the terpenoid biosynthetic pathway) from CWRs to increase resistance to biotic stress in cultivated tomatoes and other commercial plant species (Bleeker et al., 2012).

*Genetic modification* (GM) technology has been considered a revolutionary solution to transfer target genes to crop cultivars to obtain desired traits. Commercial GM crops typically produce their target product and yield as expected, and they have the advantage of not suffering from the introduction of other linked genes (linkage drag). Genetic engineering techniques are particularly useful when the desired trait is not present in the germplasm of the crop or when the trait is very difficult to improve by conventional breeding methods. A well‐known example of the use of this technology was in the production of transgenic *Bacillus thuringiensis* (Bt) crops (Tabashnik, 2010). However, there is some evidence that the use of GM technology to manipulate drought tolerance might not be as effective as traditional breeding methods (Gilbert 2014), including introgression (Honsdorf et al., 2014). In addition, the safety of foods developed from transgenic crops continues to remain a concern to the public.

Another approach to facilitate crop production is to induce mutations in existing genes rather than introduce new genes (Lusser, Parisi, Plan, & Rodriguez‐Cerezo, 2012). This approach includes cisgenesis, intragenesis, genome editing, RNA‐dependent DNA methylation, and oligo‐directed mutagenesis techniques, some of which have been used for crop improvement using CWR species (Table 2). Cisgenesis, for example, refers to the GM of crop plants with genes from the crop plant itself or from a sexually compatible donor such as a CWR. This technique has been successfully used to confer resistance to late blight in potatoes (Haverkort, Struik, Visser, & Jacobsen, 2009) and scab resistance in apples (Vanblaere et al., 2011). It is important to note that because cisgenesis only transfers a gene from a native or cross‐compatible species, this results in plants with a performance comparable to that possible from conventional breeding (Krens et al., 2015). However, transfer of a single desired gene by cisgenesis avoids any linkage drag and reduces the time involved compared with traditional breeding strategies. Although these new techniques outperform traditional breeding in some aspects and are easily adopted by the industry, their application to the production of a wide range of commercial crops depends on many factors such as the technical efficiency of some processes, the extent of social acceptance, and worldwide regulatory restrictions. Nevertheless, these technologies are useful in accelerating gene introduction and producing plants with desired traits, and could be used as an alternative strategy for crop improvement in the absence of other efficient approaches.

High‐throughput genomic approaches have become an important tool in efficient use of crop genetic resources, including CWRs, deposited in gene banks. High‐throughput genotyping of stored accessions allows the examination of genetic relationships, which enable breeders to effectively select the accessions of interest based on their genetic background (Kadam et al., 2016). Phylogenetic trees generated from these genomic data also allow the construction of core germplasm collections (Brown 1989) representing the allelic richness of the gene bank. Although large‐scale phenotyping remains time‐consuming and costly, high‐throughput phenotyping of the representative core collection for the traits of agronomic importance is less laborious than characterizing the entire collection (Honsdorf et al., 2014). Consequently, genomic‐estimated breeding values for the core collection facilitate genomic selection of superior CWR accessions (Xavier, Muir, & Rainey, 2016).

On the other hand, there is an increasing need to store and interpret datasets generated from the large‐scale characterization of wild germplasm collections (Li et al., 2014; Qi et al., 2014; Xu et al., 2012; Zhou et al., 2015). A representative example is the comprehensive database, SoyBase (http://soybase.org/), which is dedicated to soybean research especially to the breeding community. Over 40,000 genomewide SNPs for 19,652 *G. max* and *G. soja* accessions are accessible to the public (Song et al., 2015), and seeds of accessions of interest can be obtained, with certain restrictions, through the USDA Germplasm Resources Information Network (GRIN, http://www.ars-grin.gov/). The benefits from this database to the academic and breeding communities have been extensive (Kadam et al., 2016; Patil et al., 2016; Song et al., 2015; Zhang et al., 2016). Thus, integration of advanced biotechnologies and the systemic curation of germplasm banks will play an important role in the increased use of crop genetic resources, particular CWRs. All of these expectations are based on the assumption that representative CWRs are deposited in the gene banks and accessible to researchers and breeders, however, and systematic efforts are needed to ensure this.

## 4 Conservation of CWR and Accessibility to Breeders and Geneticists

There are mounting concerns about the impact of climate change, including its severe threat to biodiversity and the survival of some species, including CWRs. Consequently, international agreements have been made to ensure the security of genetic resources, and conservation strategies have been promoted to make these resources more widely available (Heywood, Casas, Ford‐Lloyd, Kell, & Maxted, 2007; Khoury, Laliberte, & Guarino, 2010; Maxted, Kell, Ford‐Lloyd, Dulloo, & Toledo, 2012; Maxted et al., 2010). In the past decade, there has been a noticeable increase in the number of publications describing the use of wild relatives for crop improvement. However, most of the wild plant species collected primarily have been used only for research rather than for long‐term conservation. Thus far, approximately 2–6% of the collections in global gene banks are CWRs (Maxted & Kell, 2009), and approximately 1.6% of those CWR species rank among the highest priorities for immediate conservation because of their importance in global food security (Maxted et al., 2012). Therefore, it is imperative that we act immediately to conserve plant wild relatives to ensure sustainable agricultural development to feed an increasing number of people.

Inventories must be made as the first step to conserve CWRs for candidate species. CWR inventories can be (and have been) made at international, national, and regional levels (Berlingeri & Crespo, 2012; Khoury et al., 2013; Landucci et al., 2014; Maxted & Kell, 2009) or can be focused on a particular crop taxon (Castaneda‐Alvarez et al., 2015; Khoury et al., 2015; Zhao et al., 2005). Currently, several of these inventories have deposited information such as taxonomic origins, regional priority categories, and proposed conservation strategies into electronic databases (Table 3). For example, South Africa, Europe, Mediterranean, Harlan, and de Wet CWR inventories have been started and are available to the public (Table 3). Many collection and inventory gaps remain (Halewood & Sood, 2006), however, and continuous efforts will be needed to fill these gaps. It is important to note that although the general guidelines for inventorying, prioritizing, and conserving CWRs have been proposed, the specific conservation strategies are regionally dependent. However, once an inventory is available, it would greatly facilitate the targeting of conservation actions. The ultimate goal should be to build a comprehensive inventory for CWRs aimed at integrating the information about CWRs dispersed among individual agencies or countries, and to make these data available globally to researchers and breeders for efficient conservation management and usage.

**Table 3:** Global and regional online portals for CWR inventories

	Database	Portal address
Global	Crop Wild Relative Global Portal	http://www.cropwildrelatives.org/
	Crop Wild Relative & Climate Change	http://www.cwrdiversity.org/
	Crop Genebank Knowledge Base	http://cropgenebank.sgrp.cgiar.org/
	Gateway to Genetic Resources	https://www.genesys-pgr.org/welcome
	Global Crop Diversity Trust	http://www.bioversityinternational.org/cwr/
	International Center for Tropical Agriculture	http://dapa.ciat.cgiar.org/
Regional	Flora of North America	http://floranorthamerica.org/
	European Cooperative Programme for Plant Genetic Resources	http://www.ecpgr.cgiar.org/
	South Africa region	http://www.cropwildrelatives.org/sadc-cwr-project/
	Europe and the Mediterranean	http://www.pgrsecure.org/
	Harlan and de Wet CWR inventory	http://www.cwrdiversity.org/

CWR, crop wild relative.

Prioritizing CWRs is important for efficient conservation efforts. For various reasons, some national or local inventories are focused only on major crops (Berlingeri & Crespo, 2012; Khoury et al., 2013; Landucci et al., 2014; Maxted & Kell, 2009; Vincent et al., 2013). In these inventories, higher priorities are given to the wild relatives falling into primary or secondary GPs over other more distant ones, as it is relatively easy to transfer traits between species within the primary GP. In a few cases, however, information on some distantly related wild species has been included, for example, wheat congeners that have been widely used in wheat improvement (Colmer et al., 2006). Prioritization of CWR species especially by the breeding community will necessarily depend on various factors such as their importance in the global food supply (wheat, rice, maize, sugarcane), nutrition (quinoa), biofuel (cassava), and their biological significance, socioeconomic value, or eco‐geographic distribution. Some CWR species growing in harsher environments should be collected because they might possess useful characteristics beneficial for crop improvement. However, a fundamental difficulty in prioritizing CWR species is that there is no complete understanding of how many CWR species even exist and where they are located. This difficulty could be gradually overcome by systematic collections in the geographic areas of prioritized CWRs proposed by Castaneda‐Alvarez et al. (2016). These authors also suggest the use of a final priority score (FPS) to prioritize CWRs, where the FPS for each CWR is based on a comprehensive assessment of its overall significance (Castaneda‐Alvarez et al., 2016). For long‐term agricultural purposes, all CWR species warrant conservation, as any CWR or wild plant species might contain useful genetic diversity beneficial for crop breeding, as previously discussed.

Crop wild relatives themselves can be conserved either in situ (in natural habitats managed as genetic reserves) or ex situ (seed, in vitro, or field gene banks) (Maxted et al., 2012). Thus far, ex situ conservation of CWRs in seed banks and gene banks has provided a convenient means of maintaining the viability of seeds for long periods of time (Barazani, Perevolotsky, & Hadas, 2008; Maxted et al., 2012; Schoen & Brown, 2001; van Slageren, 2003). However, maintaining seed samples outside their original natural habitats usually results in a decrease in genetic diversity and its associated consequences, including increased homozygosity and inbreeding depression (Schoen & Brown, 2001). In addition, storing genetic resources in seed banks freezes evolutionary processes that occur in nature. To overcome these risks, complementary ex situ and in situ approaches, with an emphasis on in situ conservation, are considered optimal to ensure the conservation of genetic resources (Maxted et al., 2012; Schoen & Brown, 2001). With integrative efforts by botanists, ecologists, and crop breeders, significant progress has been made for comprehensive CWR conservation at different levels (Khoury et al., 2013; Maxted et al., 2011; Meilleur & Hodgkin, 2004; Zhao et al., 2005) (Tables 3 and 4). If this effort is continued and increased, it should expand our knowledge of CWRs and how they might best be used in future breeding regimes.

**Table 4:** Core collections of crop wild relatives

Crop	Wild relatives	Storage location^a^
Rice	*Oryza rufipogon*,* Oryza officinalis*,* Oryza granulata*	Chinese Academy of Agriculture Sciences; International Rice Research Institute
Barley	*Hordeum spontaneum* and other *Hordeum* wild species	International Barley Core Collection (~300); USDA‐ARS National Small Grains Collection
Wheat	*Triticum*,* Aegilops*,* Dasypyrum villosum*	The Wheat Genetics Resource Center (14,000)
Soybean	*Glycine soja*	USDA Soybean Germplasm Collection (1,100); Chinese National Crop Genebank (6,172)
Sorghum	23 wild *Sorghum* species	International Crops Research Institute for the Semi‐Arid Tropics (449)
Tomato	Wild *Lycopersicon* and *Solanum* species	Tomato Genetics Resource Center (1,196)
Potato	187 wild *Solanum* species	International Potato Center

a Brackets give the number of conserved wild relatives (accessions or species) for each crop.

Accessibility of CWR resources to researchers and breeders is extremely important for continued utilization of CWRs for crop improvement. Knowledge of the genetic diversity present in a germplasm bank is critical for its potential exploitation by breeders. Thus far, many international collections such as CIAT, Crop Trust, and Kew, as well as national gene banks such as the US germplasm resource (http://www.ars-grin.gov/), have efficiently conserved a number of CWR species and provided information such as prebreeding data, geographic distribution, and potentially useful traits for these CWRs (Table 3). However, conservation of underrepresented CWRs and insufficient information for stored CWR species remain the greatest obstacles for their use in plant breeding. A preliminary field survey might be most helpful in deciding whether a certain CWR species has a direct use for crop improvement.

Further, an information system for gene banks containing comprehensive and up‐to‐date data that are accessible to breeders is also critical to tracking accessions for management purposes. Thus far, a number of international or regional information databases exist for CWR species (Table 3), with some degree of accession‐level data for breeding utilization. In addition, sharing of reports from prebreeding tests and field performances of CWR species conducted by breeders would be helpful. Regular monitoring and updating of the conservation status of CWR species would also greatly assist in closing the collection–conservation gaps.

Because we know very little about the molecular genetic diversity for most CWR taxa, Castaneda‐Alvarez et al. (2016) have suggested that we use geographic and ecological criteria to determine their conservation status. This strategy thus uses eco‐geographic representativeness to evaluate the true adaptive ability of CWR taxa to specific conditions. This approach is consistent with the use of CWRs to develop improved crop varieties that are better adapted to various combinations of biotic and abiotic environmental conditions.

## 5 Conclusions and Perspectives

The potential of the genetic diversity stored in wild species banks for use in crop improvement appears to be much greater than we previously imagined. Recent increases in the use of wild resources have occurred because of the recognition of the usefulness of CWRs for food security and the development of advanced biotechnologies (Honsdorf et al., 2014; Langridge & Fleury, 2011). The examples reviewed here and in other studies (Brozynska et al., 2015; Colmer et al., 2006; Ford‐Lloyd et al., 2011; Hajjar & Hodgkin, 2007; Maxted & Kell, 2009; Nevo & Chen, 2010; Zamir, 2001) demonstrate that there is a wealth of genetic diversity retained in wild relatives of various crops, much of which remains to be explored.

**Figure 2:**
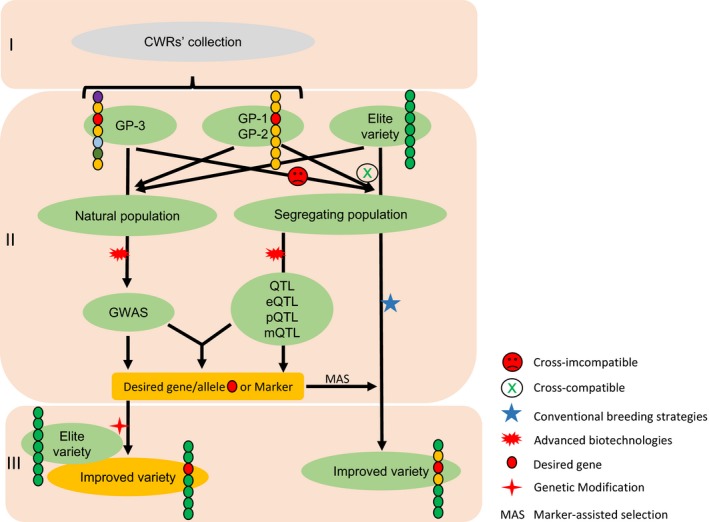
Flowchart showing the application of crop wild relatives (CWRs) and advanced technologies in crop improvement. Stage I, CWR collections. Stage II, gene discovery. Advanced biotechnologies can facilitate identification of desired genes or markers in CWRs using genome‐wide association studies (GWAS) and quantitative trait loci (QTL) strategies. Stage III, gene transfer. The resultant markers and causal genes can be transferred to crops by conventional breeding programs and/or transgenic techniques

Rapid progress of advanced biotechnologies that can bridge genotype–phenotype gaps will facilitate the use of CWRs for crop improvement. Thus far, a number of QTL and SNPs associated with agronomically and ecologically important traits have been identified in wild species by linkage analyses, GWAS, and combined analyses of “omics” approaches and linkage mapping (Table 1; Figure 2). The rapid improvement of biotechnological tools, such as diverse omics approaches, has resulted in promising advances and no doubt will become routine in plant breeding programs. Advanced biotechnologies, such as genome editing and cisgenesis/intragenesis, are continuously being developed and will accelerate the conservation and use of genetic diversity retained in CWRs, resulting in agriculture sustainability. Collection and conservation of prioritized CWRs could be intensively conducted in the geographic regions harboring the greatest richness of taxa (Castaneda‐Alvarez et al., 2016). Instead of focusing on a single beneficial trait, the overall genetic provenance and adaptive value in each CWR species should also be taken into account to prioritize CWRs and guide efficient and effective CWR conservation. Knowledge of the conservation (in situ and ex situ) status of CWRs will continuously increase by conservation gap analysis using geographic and ecological variation metrics as a proxy, maximizing the efficiency of conservation actions. Global initiatives (Dempewolf et al., 2014; Vincent et al., 2013) have increased CWR conservation efforts and should be complemented by regional and national actions (Meilleur & Hodgkin, 2004). Collaborations between local institutions or organizations can help to build agreements about the effectiveness of in situ or ex situ conservation, and foster sharing of wild resources. International CWR exchanges and/or introductions could also greatly benefit the extensive conservation and utilization of CWRs.
