# Supplementary figures and images for: Back into the wild—Apply untapped genetic diversity of wild relatives for crop improvement
Source: Evol Appl. 2016 Dec 10;10(1):5–24. doi: 10.1111/eva.12434 (PMC5192947; doi:10.1111/eva.12434)

(A)

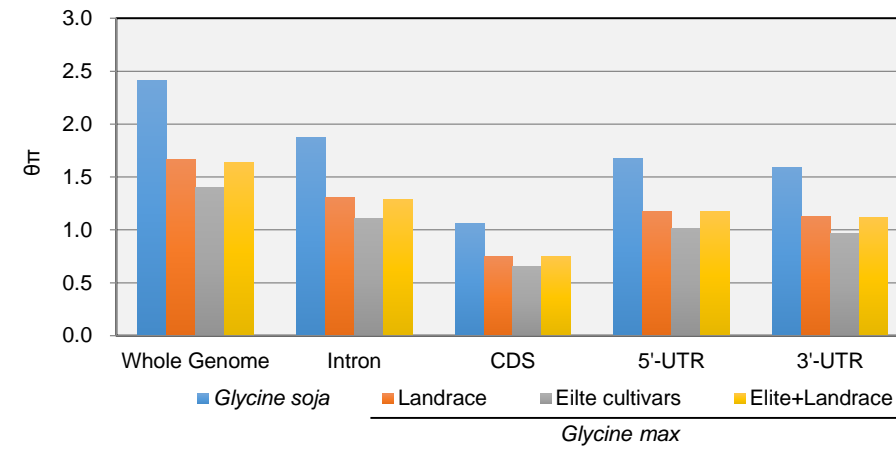

(B)

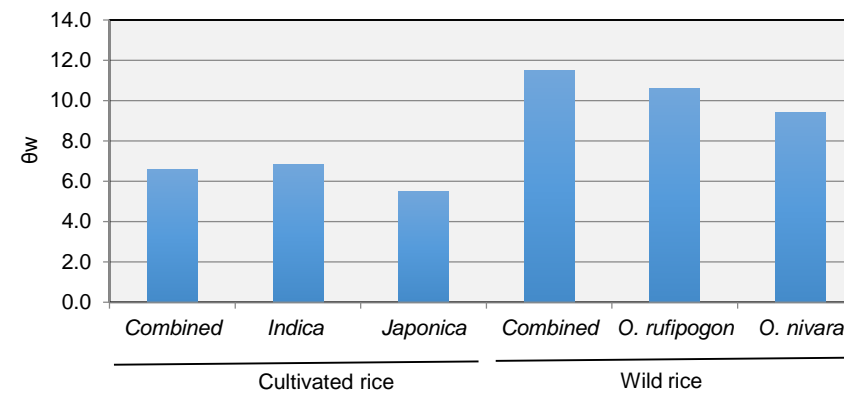

Supplement: Supplementary file 1 [file EVA-10-5-s001.pdf]
